# Contributions of multimodal imaging

**DOI:** 10.1186/1470-7330-14-S1-O40

**Published:** 2014-10-09

**Authors:** Wim JG Oyen

**Affiliations:** 1 Department of Radiology and Nuclear Medicine, Radboud University Medical Center, Nijmegen, The Netherlands

## 

Imaging for radiotherapy is indicated during all phases of diagnosis and treatment: for staging, radiotherapy planning, therapy response monitoring and prediction, follow-up and relapse detection. Besides conventional CT imaging, functional (e.g. MRI) and molecular (e.g. PET) imaging modalities are increasingly used in patients before, during and after radiotherapy.

## Staging and radiotherapy planning

Given the limitations of CT for adequate detection of lesions in many tumor types, functional and molecular imaging is used for improved detection of lesions and assessment of loco-regional disease that should be included in the radiation treatment plan [1,2]. This may result in an increase as well as a decrease of the gross tumor volume (GTV). Increasing the GTV with lesions suspicious of malignancy enhances the likelihood of optimal tumor control. Conversely, exclusion of lesions detected on CT, but with a low likelihood of malignancy on MRI or PET decreases the GTV and thus the side effects associated with larger fields while maintaining optimal loco-regional control of the cancer [3].

## Therapy response prediction and monitoring

In case radiotherapy is indicated after previous systemic therapy (e.g. in malignant lymphoma), molecular imaging is increasingly used to assess areas of viable tumor, which may prevent the need to irradiate large areas of residual abnormalities which contain no viable tumor [4].

Furthermore, adaptation of treatment during radiotherapy (before the full radiation dose has been administered) offers the chance to increase the dose to radioresistant areas in solid tumor, while the dose to areas that respond very well may be decreased [5]. The clinical impact of this paradigm is currently the subject of many clinical trials, which will make radiation therapy a much more dynamic treatment by a more or less continuous evaluation of tumors by functional and molecular imaging and subsequent adaptation of radiotherapy plans. This concept will be largely facilitated by the use of MRI-Linac and maybe in future PET-Linac.

## Follow-up and relapse detection

In case of suspicion of relapse, advanced imaging may preselect those patients who are potentially eligible for salvage therapy, but require invasive diagnostic procedures to establish a histological diagnosis. With an increasing number of local and systemic treatment options becoming available for many tumor types, the demands for optimal follow-up becomes increasingly important [6]. The choice for the optimal imaging modality and the timing of imaging are crucial for adequately restaging patients. The impact of radiotherapy-related effects such as radiation-induced inflammation and fibrosis on the accuracy of imaging procedures has to be considered. When considering imaging during follow-up of patients, the clinical need for early detection and the availability of meaningful subsequent treatment should be weighed against the increasing costs associated with the frequent use of advanced imaging techniques (i.e. appropriate use).

## Conclusions

Multimodal advanced functional and molecular imaging is increasingly used before, during and after radiotherapy. The evidence for many indications is rapidly evolving. This offers great potential for optimized, individualized treatment of patients, providing better chances for loco-regional tumor control and less treatment-associated side effects.
